# Protection of the human gene research literature from contract cheating organizations known as research paper mills

**DOI:** 10.1093/nar/gkac1139

**Published:** 2022-12-07

**Authors:** Jennifer A Byrne, Yasunori Park, Reese A K Richardson, Pranujan Pathmendra, Mengyi Sun, Thomas Stoeger

**Affiliations:** School of Medical Sciences, Faculty of Medicine and Health, The University of Sydney, NSW, Australia; NSW Health Statewide Biobank, NSW Health Pathology, Camperdown, NSW, Australia; School of Medical Sciences, Faculty of Medicine and Health, The University of Sydney, NSW, Australia; Department of Chemical and Biological Engineering, Northwestern University, Evanston, USA; School of Medical Sciences, Faculty of Medicine and Health, The University of Sydney, NSW, Australia; Department of Chemical and Biological Engineering, Northwestern University, Evanston, USA; Department of Chemical and Biological Engineering, Northwestern University, Evanston, USA; Successful Clinical Response in Pneumonia Therapy (SCRIPT) Systems Biology Center, Northwestern University, Evanston, USA; Center for Genetic Medicine, Northwestern University School of Medicine, Chicago, USA

## Abstract

Human gene research generates new biology insights with translational potential, yet few studies have considered the health of the human gene literature. The accessibility of human genes for targeted research, combined with unreasonable publication pressures and recent developments in scholarly publishing, may have created a market for low-quality or fraudulent human gene research articles, including articles produced by contract cheating organizations known as paper mills. This review summarises the evidence that paper mills contribute to the human gene research literature at scale and outlines why targeted gene research may be particularly vulnerable to systematic research fraud. To raise awareness of targeted gene research from paper mills, we highlight features of problematic manuscripts and publications that can be detected by gene researchers and/or journal staff. As improved awareness and detection could drive the further evolution of paper mill-supported publications, we also propose changes to academic publishing to more effectively deter and correct problematic publications at scale. In summary, the threat of paper mill-supported gene research highlights the need for all researchers to approach the literature with a more critical mindset, and demand publications that are underpinned by plausible research justifications, rigorous experiments and fully transparent reporting.

## INTRODUCTION

Irreproducible research represents a major problem that hinders research translation and generates damaging levels of research waste (1–4). There are many causes of irreproducible research results (1–6), but relatively few studies have focussed upon the contributions of research fraud (7,8). This review will describe problems affecting the human gene research literature through possible contributions from contract-cheating organisations known as research paper mills, which provide undeclared services to support research manuscripts and publications (8–12).

The review will provide a brief introduction to human gene research and then introduce research paper mills. The concept that scientific articles can be bought and sold (8–12) contradicts everything that most researchers wish to believe about scientific publishing, and some readers may doubt that paper mills exist. We will therefore summarise what is known or predicted of paper mill operations and their likely drivers and enablers. We will summarise the evidence that paper mills have contributed to the human gene research literature at scale and describe why human gene research may be particularly vulnerable to industrial-scale research fraud. Finally, we will describe how researchers (including scientific editors and peer reviewers) and publisher staff can recognise problematic gene research manuscripts and publications. As the improved detection of problematic research could drive the evolution of paper mill manuscripts, we will also propose approaches to deter and correct problematic gene research at scale.

## CHALLENGES IN STUDYING HUMAN GENES

**Figure 1.**
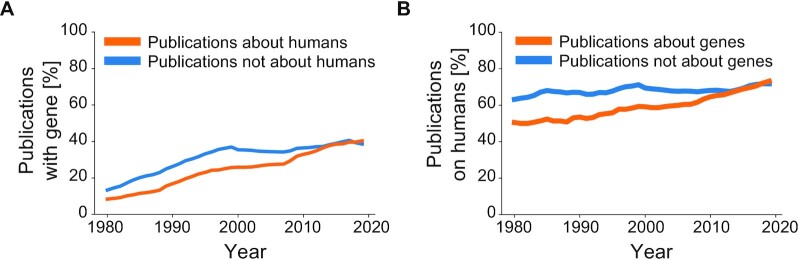
A substantial share of the biomedical literature from 1980–2020 features genes or their molecular products. (**A**) Percentages (Y axis) of PubMed publications per year (X axis) that feature at least one gene, according to PubTator (105). Percentages show whether publications are about humans, according to their MeSH terms (orange, about humans; blue, not about humans). (**B**) Percentages (Y axis) of PubMed publications per year (X axis) that are about humans, according to their MeSH terms. Percentages show publications that do/ do not feature at least one gene, according to PubTator (105) (orange, features at least one gene; blue, does not feature any gene).

**Figure 2.**
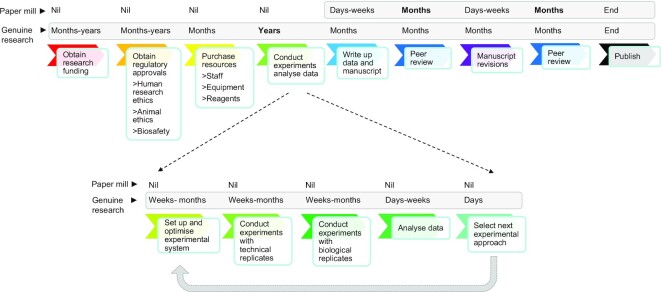
Diagrammatic comparison of the steps required to publish genuine targeted human gene research versus those required to publish fabricated human gene research by paper mills. Most steps also pertain to gene research in other organisms, with the exception of human research ethics approval. Steps are shown in typical chronological order from left to right. Steps required for laboratory experimentation are expanded in the lower panel. Estimates of time periods required to complete each step (nil, days, weeks, months, years) by either genuine researchers or by paper mills (indicated at left) are shown above each diagram. ‘Nil’ indicates steps that do not need to be conducted by paper mills. Time estimates may not reflect the requirements of individual projects, and some steps can be undertaken simultaneously, such as obtaining regulatory approvals and purchasing equipment and/or reagents. Rate-limiting steps for genuine researchers or paper mills are highlighted in bold.

Over the last 50 years, biomedical research became increasingly focused on genes and their encoded transcripts and proteins (13), where in recent years, 40% of all publications in PubMed have featured at least one gene (Figure 1). Prior to the late 1990s, experimental techniques were suitable for studying single or small numbers of human genes, which reflected a research landscape where only a minority of human genes had been experimentally identified (13). In the present day, practically all human genes have been identified (14) and it is theoretically possible to investigate any human gene through tailored reagents. Despite the availability of ∼40 000 human genes for research (14), both the experimental analysis of single or small collections of genes (which we will refer to as ‘targeted gene research’) and research at the genome-wide scale remains difficult, expensive and slow. In the case of targeted gene research, which will be the focus of this review, time and resources are required to obtain the necessary funding and regulatory approvals and then to optimize, conduct, repeat, analyze and write up the results of laboratory experiments (Figure 2).

**Figure 3.**
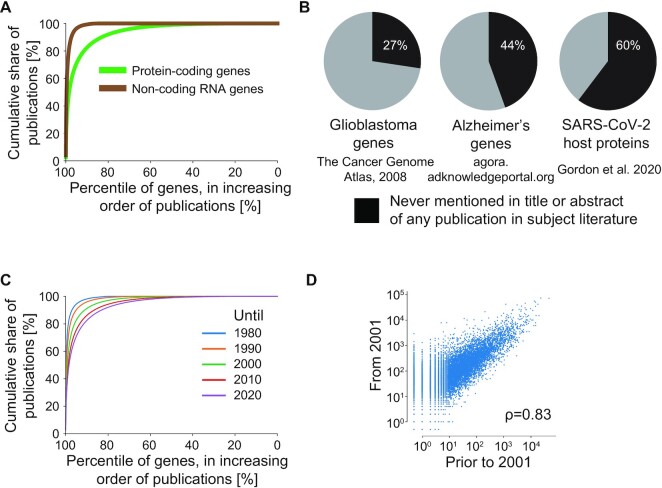
Scholarship distributes unevenly across genes, and knowledge gaps persist in important areas of research. (A, B) Genes are ranked (percentiles, X axis) according to the numbers of mentions in titles and abstracts, where the 100- and 0-percentiles correspond to the most/ least frequently investigated gene, respectively. (**A**) Cumulative share (percentages, Y axis) of all mentions of protein-coding (green) and non-coding RNA genes (brown) within titles and abstracts of PubMed publications on humans according to MESH terms. (**B**) Cumulative share (percentages, Y axis) of all mentions of protein-coding genes within titles and abstracts of PubMed publications on humans according to MESH terms, until the indicated years. (**C**) Percentages (black shading) of protein-coding genes identified by the indicated studies (106, https://agora.adknowledgeportal.org, 107) that have never appeared in the title or abstract of any publication in the relevant literature (MeSH term on Glioblastoma, MeSH term on Alzheimer's disease, LitCOVID (108)). Genes in species other than humans were mapped to their orthologs via HomoloGene. (**D**) Numbers of PubMed publications since 2001 (Y axis) or prior to 2001 (X axis) that mention at least one human protein-coding gene (blue dots) in the title or abstract. The year 2001 reflects the publication of the first draft of the human genome (24,25). ρ indicates Spearman's rank correlation coefficient. Protein-coding genes that are offset from the X axis have never been mentioned in any title or abstract.

Beyond practical considerations, human gene research is complicated by the number of individual genes that are available for study, and the variable levels of research attention that different genes have received (13,15–23). Although the first draft of the human genome (24,25) was expected to increase the types and numbers of human genes that were subjected to experimentation, this broadening of research focus did not occur (Figure 3) (13,15–17,20,23). Instead, well-characterized genes continue to be preferentially studied, due to reagent availability, researcher unwillingness or difficulties in changing research focus, and possible career penalties for investigating under-studied human genes (17,20,23). Indeed, the rate at which new protein-coding genes have entered the literature has been in decline since 2000 (23).

The resulting uneven human gene research landscape (Figure 3) has many consequences. Researchers know a great deal about few protein-coding genes and even fewer ncRNAs (23), yet very little about the majority (Figure 3), where gene knowledge may not be proportional to biomedical significance (15–17,19). Many publications about well-studied genes can lead to information overflow, where not all literature can be read, synthesized, or used (26). In contrast, the scant research attention given to most human genes creates many knowledge gaps (27), which can be challenging to address due to limited reagents and funding (17,22) and incorrect gene annotations (28). In summary, many human genes, combined with a highly uneven human gene research landscape (Figure 3), create significant challenges for information synthesis and the identification of productive areas for future gene research (16,17).

## INTRODUCTION TO RESEARCH PAPER MILLS

The term ‘paper mill’ was first employed in the 1970s (29,30) to describe commercial organizations that sell essays and theses to high school, undergraduate and/or postgraduate students. These organizations are now referred to as ‘essay mills’ (31), and ‘paper mill’ is applied to commercial entities that (also) sell undeclared services in relation to research manuscripts and publications (8–12,32,33). While recognizing that very little empirical research has been published about paper mills, we will summarize the literature that describes or predicts elements of paper mill operations and their products and services.

Paper mills are alleged to operate in many countries and to offer a variety of questionable and/or illegal services to a range of clients across different research disciplines (8–10,32,33). Clients of paper mills are likely to be academics, research students and clinicians who do not have the time, facilities and/or training to conduct the genuine research that is required of them (10,11,32,34,35). Paper mills are alleged to sell author slots on accepted manuscripts (9), where the price can depend upon authorship position (32,33), as well as research datasets that clients can insert into manuscripts (34) and/or entire manuscripts that can be authored by teams as required (32,35–37). It is predicted that paper mill manuscripts that describe laboratory research will frequently include falsified or fabricated experimental results, due to genuine laboratory research being difficult, expensive and slow (10,27,32,36,38).

Paper mill employees are also alleged to undertake manuscript submission, which can involve the simultaneous submission of manuscripts to multiple journals, presumably to increase the likelihood and speed of manuscript acceptance (10,32). Journals that accept suggestions of recommended peer reviewers can be provided with falsified reviewer names and contact details (8,35). Paper mill employees may also direct communications in response to PubPeer and other post-publication notifications (36,37,39) and offer added value to their publications through citations in subsequent manuscripts (37), which could benefit both paper mill clients and journals. The cost of paper mill services is likely to depend on the extent of services supplied (32,33), which may in turn depend upon the requirements of the targeted journal or journal category.

As many activities of covert businesses resemble those of overt or legal companies (40,41), some features of paper mill operations can be predicted from those of genuine research support services. Paper mills are likely to maximize profits by generating plausible research manuscripts as quickly and cheaply as possible (27,38). A variety of operating models could support this requirement (42). Paper mills could range in size from single individuals to large teams that provide a broader range of services to more clients (32). Paper mills could operate within businesses such as academic editing, biotechnology and/or contract research companies (32,36). Associations with apparently legitimate businesses could provide long-term concealment (40,41), as well as access to staff whose expertise and knowledge meet the expectations of clients and targeted journals.

## KEY DRIVERS AND ENABLERS OF PAPER MILLS

Clients are believed to be drawn to paper mills by imbalances between the pressures and opportunities to publish research in different settings (8,10,11,32,35,43). While publication expectations can provide research support incentives and encourage the timely dissemination of results (44), publication requirements or quotas are more problematic. Some researchers describe publication quotas to achieve or retain career positions, where quotas are not matched by available research time, training, funding, infrastructure, personnel and/or language support (32,35,43). Hospital-based clinicians may be particularly vulnerable to publication quotas (34,35,45,46), as their time, training and resources are directed towards patient care as opposed to research (47). Cash rewards for publications can also drive the use of paper mills, by providing funds to pay for paper mill services (32,48).

Paper mills also benefit from recent developments in academic publishing. The growth of online journals that require author publication fees may be driving an increasingly profit-based publishing model (49). Digital publishing also allows more manuscripts to be published more quickly and enables the creation of new journals whose scope may overlap with or duplicate that of existing journals (5,26,27). While the creation of new journals is clearly important to support new or expanding fields, this can lead to journal oversupply in fields where manuscript numbers are more stable. Growing manuscript numbers also create significant challenges for peer view (8,50,51), where insufficient numbers of over-burdened peer reviewers enable the publication of poor quality manuscripts, including those from paper mills.

Finally, the mismatch between journals’ capacity to publish manuscripts and achieve timely post-publication corrections is a significant enabler of low-quality and fraudulent research (39). The stigma that can surround post-publication corrections is likely to reinforce perceptions that published errors and research fraud are infrequent, of little consequence, and/or will be addressed by science's self-correction capacity (5,37,52,53). Furthermore, retractions and corrections generate no publisher income and few citations, and hence may not be prioritized by some journals or publishers. In cases where post-publication concerns are investigated, outcomes can be delayed by the need to receive responses from authors and/or institutions, where there may be few incentives for timely communications (39). In summary, the current inability to achieve timely corrections of the published literature at scale specifically disadvantages genuine research, while providing an invaluable asset for paper mills (10).

## EVIDENCE THAT PAPER MILLS CONTRIBUTE TO THE HUMAN GENE RESEARCH LITERATURE

Research paper mills were brought to international attention by Hvistendahl in 2013 (9), who described paper mills that offered authorship slots and entire manuscripts for sale. The possibility that paper mills might be targeting human genes was then proposed by Byrne and Labbé in 2017 (38). These authors identified examples of strikingly similar, formulaic papers that reported the effects of knocking down individual human genes in cancer cell lines (38). The retraction notice for one paper stated that the experiments had been outsourced to a biotechnology company, representing a link between a problematic gene research paper and undeclared external support (38,39).

Many of the papers reported by Byrne and Labbé (38) were found to share identical incorrect ‘non-targeting’ shRNA sequences that were instead predicted to target human genes. Some papers also described targeting reagents that were verified to target different human genes from those claimed (38). While hundreds of gene research papers that describe incorrect non-targeting gene knockdown controls are indexed by Google Scholar, authors and/or reagent supply companies have only explained or corrected a small fraction of these papers (39). Although few corrected papers may reflect well-recognised barriers to post-publication correction (52,53), the lack of corrections to many papers with identical errors could also reflect the unwillingness of paper mills to draw critical attention to their products (39).

Wrongly identified nucleotide sequence reagents in gene research papers led to the creation of the semi-automated Seek & Blastn tool (54), which fact-checks the claimed identities of human nucleotide sequence reagents using Blastn (55). Seek & Blastn has since been applied to over 11,700 articles across both targeted and journal corpora (56). Seek & Blastn screening supported by manual verification of reagent identities found 712 articles with wrongly identified sequences that were published across 78 journals (56). Most of the 1,535 wrongly identified nucleotide sequences represented claimed targeting reagents for the analysis of 365 human protein-coding genes and 120 non-coding RNAs (56). Although wrongly identified nucleotide sequences can arise through honest error, many problematic articles identified by Park *et al.* contained implausible errors, such as claimed human gene targeting reagents with either no identifiable human gene target or targeting reagents that were predicted to target orthologous genes in other species (56).

A growing number of article retractions also reflect the activities of paper mills. In 2017, the journal *Tumor Biology* retracted 106 articles published from 2012–2016 in response to evidence of manipulated peer review, where almost all (92%, 98/106) retracted papers analysed single or small groups of human genes (57). A further 250 retracted articles were described by Qi *et al.* (58), including many human gene research articles published across 19 journals. Subsequent interviews with the authors of some retracted articles (58) described the undeclared use of publication agencies or paper mills (35).

While recognising that the reason(s) for article retraction are not always disclosed (39,59), in March 2022, we searched the literature and the Retraction Watch database (http://retractiondatabase.org/) (60) for cancer research papers that have been retracted due to paper mill involvement. We searched the literature indexed by Google Scholar using the search terms ‘retraction’ AND (‘paper mill’ OR ‘company’) AND ‘cancer’. The Retraction Watch database was filtered for the category ‘(BLS) Biology - Cancer;’ under the column ‘Subject’, and for ‘+Paper Mill;’ or ‘+Concerns/Issues about Third Party Involvement;’ under the column ‘Reason’. All identified retraction notices were then manually screened for the term ‘paper mill’, or references to the undisclosed use of any third party and/or sale of article. These combined search strategies identified 204 retraction notices that were published across 36 journals between 2016 and 2021 (Table 1), where 167/204 (81.9%) retracted papers referred to human genes in their titles. These results are similar to those described by a recent scoping review that identified over 300 retracted articles due to suspected paper mill operations, where ‘mir’ (miRNA) represented the most frequently identified keyword (61).

**Table 1.** Summary of retraction notices that referred to use of a paper mill, the previously undisclosed use of any third party and/or sale of article

Retractions referring to a paper mill, previously undisclosed use of third-party and/or sale of article (*n*)			204
Unique journals (*n*)			36
Unique publishers (*n*)			14
Publication year of article (median (range))			2019 (2013–2021)
Publication year of retraction notice (median (range))			2021 (2016–2022)
Retracted articles from China (proportion (%))			204/204 (100%)
Retracted articles affiliated with hospitals in China (proportion (%))			186/204 (91.1%)
Retraction notices that were included in mass retractions (proportion (%))			151/204 (74.0%)
Retracted articles that had studied human gene(s) (proportion (%))			167/204 (81.9%)
**Journal** ^a^	**Publisher**	**Journal impact factor** ^b^	***n* (%)**
RSC Advances	Royal Society of Chemistry	3.361	68 (33.3%)
Journal of Cellular Biochemistry	Wiley	4.429	45 (22.1%)
International Journal of Immunopathology and Pharmacology	Sage Publications	3.219	16 (7.8%)
Cancer Biotherapy & Radiopharmaceuticals	Mary Ann Liebert	3.099	12 (5.9%)
Technology in Cancer Research & Treatment	Sage Publications	3.399	12 (5.9%)
Journal of Materials Chemistry A	Royal Society of Chemistry	12.732	6 (2.9%)
Cellular Physiology and Biochemistry	Karger	5.5	5 (2.5%)
Naunyn-Schmiedeberg's Archives of Pharmacology	Springer Nature	3	5 (2.5%)
PLoS One	Public Library of Science	3.24	4 (2.0%)
Journal of International Medical Research	Sage Publications	1.671	3 (1.5%)
BioMed Research International	Hindawi	3.411	2 (1.0%)
Genetic Testing and Molecular Biomarkers	Mary Ann Liebert	1.795	2 (1.0%)
Acta Pharmacologica Sinica	Springer Nature	6.15	1 (0.5%)
American Journal of Translational Research	E-Century Publishing Corporation	4.06	1 (0.5%)
Bioscience Reports	Portland Press Ltd	3.84	1 (0.5%)
Cancer Biomarkers	IOS Press	4.388	1 (0.5%)
Cancer Cell International	BMC (Springer Nature)	5.722	1 (0.5%)
Cancer Chemotherapy and Pharmacology	Springer Nature	3.333	1 (0.5%)
Catalysis Science and Technology	Royal Society of Chemistry	6.119	1 (0.5%)
DNA and Cell Biology	Mary Ann Liebert	3.311	1 (0.5%)
Food & Function	Royal Society of Chemistry	5.396	1 (0.5%)
Gene	Elsevier	3.688	1 (0.5%)
Human Gene Therapy	Mary Ann Liebert	5.695	1 (0.5%)
International Immunopharmacology	Elsevier	4.932	1 (0.5%)
International Journal of Clinical and Experimental Medicine	E-Century Publishing Corporation	0.05	1 (0.5%)
International Journal of Molecular Medicine	Spandidos Publications Ltd	4.101	1 (0.5%)
Journal of Cellular and Molecular Medicine	Wiley	5.31	1 (0.5%)
Journal of Molecular Liquids	Elsevier	6.165	1 (0.5%)
Journal of Translational Medicine	BMC (Springer Nature)	5.531	1 (0.5%)
Molecular Breeding	Springer Nature	2.589	1 (0.5%)
Nanoscale	Royal Society of Chemistry	7.79	1 (0.5%)
Oncology Reports	Spandidos Publications Ltd	3.906	1 (0.5%)
Oncotarget	Impact Journals LLC	5.168	1 (0.5%)
Pharmacology	Karger	2.547	1 (0.5%)
RSC Medicinal Chemistry	Royal Society of Chemistry	N/A	1 (0.5%)
Small	Wiley	13.281	1 (0.5%)

^a^Journals are ranked according to numbers of retractions and then in alphabetic order.

^b^Journal Impact Factor is the most recent available. N/A = no journal impact factor available.

At least 11 journals across 8 publishers have now recognized the threat of paper mills through journal editorials (36,62–73). Most of these journals publish human gene research and some have specifically recognized paper mill articles that target human genes (36,65,69,73). These editorials have described features of suspected paper mill articles, either through editors’ own experiences and/or by summarizing features described elsewhere (Table 2). Repeatedly described features include manuscripts that do not fit stated journal criteria or special issue scope (63,66), conserved structures suggesting the use of manuscript templates (66,70,71), manipulated or duplicated images and figures (36,64–67,69,71), and failure to provide convincing raw data upon request (36,62) (Table 2). Some editorials also described decisions to retract multiple papers due to paper mill involvement (36,66,68,73).

**Table 2.** Problematic features relevant to human gene research that can be noted by researchers (including scientific editors and peer reviewers) and/or publisher and journal staff

		Feature can be noted by		
Key considerations	Features of concern	Researchers including scientific editors, peer reviewers?	Publisher/ editorial staff?	References
Plausibility	Title or topic outside journal/ special issue scope	✓^a^	**✓✓**	63, 66
	Inadequate justification of research topic and gene(s) and/or protein(s) analysed, such as novelty-based justification	**✓✓** ^b^		10, 71, 92
	Scope/ volume of experimental work unreasonably broad/ extensive, relative to research justification	**✓✓**		8, 90
	Gene and/or protein expression data inconsistent with previous studies	**✓✓**		89, 91, 92
	Implausible data distributions, lack of data outliers	**✓✓**		32, 73
	Highly conserved manuscript/ publication structure shared by other publications that lack common authorship	✓	**✓✓**	32, 38, 56, 66, 70, 71, 85, 86
	No/ questionable funding support listed	✓	**✓✓**	8, 36, 66, 70, 71
Transparency	Experimental procedures, reagents and/or results not identified or transparently described	**✓✓**		32, 89, 90, 92, 104
	Human research ethics/ animal care and ethics /biosafety clearance information not provided	✓	**✓✓**	8, 32, 104
	Failure to provide convincing raw data on request		**✓✓**	36, 37, 62
Accuracy	Wrongly identified reagents, eg siRNAs, shRNAs, (RT)-PCR primers, CRISPR guide sequences	**✓✓**	✓	38, 39, 54, 56
	Misidentified or contaminated cell line models	**✓✓**	✓	10, 97
	Duplicated/ manipulated images and/or figures		**✓✓**	32, 36, 37, 64, 65, 66, 67, 69, 71, 104
	Incorrect use of experimental controls	**✓✓**		38, 39, 54, 56, 90, 92

^a^✓ = feature detection possible.

^b^ **✓✓** = feature detection likely as aligned with expertise.

## WHY HUMAN GENE RESEARCH COULD BE PARTICULARLY VULNERABLE TO PAPER MILLS

Retrospective literature analyses (38,39,54,56), combined with qualitative research conducted with authors (35) and investigations led by publishers or journal editors (36,65,69,73), commonly suggest that human gene research is being targeted by paper mills. While recognising the limited empirical research conducted to date, we will consider the factors that could render human gene research vulnerable to paper mills, to predict the potential scale of this problem.

Targeted gene research may represent an attractive topic for paper mills because the associated experimental results are easy to fabricate (Figure 2). In contrast to the fabrication of genome-wide research that has been estimated to require similar effort as the acquisition of genuine data (74), targeted gene research is easier to fabricate than to produce through genuine effort (27,38) (Figure 2). Targeted gene research uses experimental techniques that generate small individual datasets that are easy to invent, with access to only basic text and image processing software. This could allow the rapid creation of targeted gene research manuscripts at prices that clients can afford (38). The use of established, widely-used experimental techniques could also allow paper mills to easily source writers with relevant expertise.

**Figure 4.**
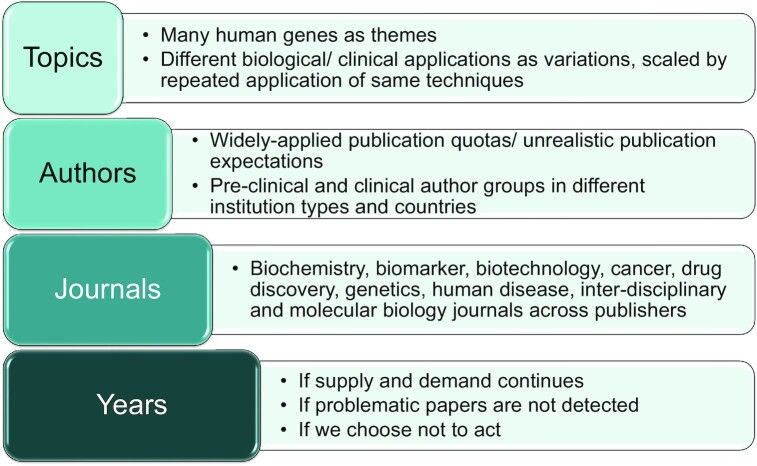
Diagrammatic representation of the many topics, many authors, many journals and many years model that could scale production and publication of paper mill contributions to the human gene research literature.

Paper mills are also likely to value topics that allow the creation of many individual manuscripts at scale (27). Targeted human gene research provides several scaling factors that could enable the production of many individual manuscripts (10,27,56) (Figure 4). Many individual human genes operating within complex regulatory networks could provide many individual research topics that can be exploited. Inconsistent gene nomenclature (75) and opaque numeric ncRNA and circular RNA identifiers (56,76) further add to the apparent numbers of human genes and transcripts that can be studied and combined. Individual genes or groups of genes can also be plausibly studied in different biological or clinical contexts (Figure 4) (27,56). Targeted gene research in the context of human cancer provides further scaling opportunities, as genes can be repeatedly investigated using the same accessible techniques (27) to examine widely-understood cancer hallmarks that are potentially relevant to many different cancer types (77,78).

The production of many individual manuscripts by paper mills will then benefit from multiple distribution or dilution factors. For example, large numbers of problematic or fabricated manuscripts require many different authors for distribution (Figure 4), as highly similar manuscripts and publications by the same authors is a recognized feature of questionable research (27). Gene research manuscripts may therefore be attractive to paper mills as they can be plausibly authored by researchers ranging from basic scientists to clinicians, who can be affiliated with different institution types in many countries (56). Unreasonable pressures to publish that are applied across large trainee, researcher and clinician populations could therefore generate many potential authors for human gene research manuscripts from paper mills.

Many gene research manuscripts produced by paper mills will also require many journals for publication, to avoid concentrations of manuscripts at individual journals that could facilitate their detection (27,36,66–73). Many journals are available to publish human gene research (Figure 4), including specialty journals in fields ranging from biochemistry to human diseases, as well as inter-disciplinary mega-journals. Different publishers also offer gene research journals across a range of journal impact factors that could appeal to a broader range of paper mill clients. Scaling the production of human gene research manuscripts is likely to exceed the reviewing capacity of the peer review community, which could further enable publication of superficially plausible manuscripts (27).

The final scaling and distribution factor that is available to paper mills is time (Figure 4). Given the absence of effective detection methods and responses, at least some paper mills may have been operating with few impediments for at least the past decade (42). The opportunity to learn over time could allow paper mills to progressively refine their business models and render their manuscripts increasingly plausible and resistant to detection. The passage of time could therefore allow paper mills to exploit new or emerging gene research topics and techniques, engage with new client types and/or publish in new journals (56).

## POTENTIAL CONSEQUENCES OF PAPER MILL CONTRIBUTIONS TO THE GENE RESEARCH LITERATURE

Gene research papers from paper mills have the capacity to derail both scientific progress and research career progression. The researchers who seem most likely to be directly affected by problematic gene research papers are those who conduct targeted gene research. Papers that describe interesting phenotypes from the repeated analysis of individual genes could encourage further laboratory research (27), where trainees, early career researchers and technical staff appear most likely to attempt to reproduce experiments, possibly with wrongly identified reagents. Failures to reproduce experimental results are then unlikely to be reported, due to the strong literature bias towards positive results (6,79). Pursuing fabricated gene research could therefore delay career progress at critical times when researchers need to be productive. Senior scientists could also be impacted by lost productivity of team members and the waste of grant funds on reagents and experiments.

As research publications tend to evolve towards describing increasing quantities of data (26,43), paper mill products could also evolve from comparatively simple manuscripts to those of greater complexity. Such publications could study more individual genes using increasingly sophisticated experimental techniques. Fabricated gene research papers that appear to describe increasing numbers of complex experiments therefore risk artificially inflating both manuscript standards and research productivity expectations. Artificially inflated expectations of targeted gene research could be increasingly difficult to achieve, which could place some gene researchers at increasing disadvantage.

Large numbers of problematic gene research papers will also affect downstream users of the gene research literature. Human gene research publications with wrongly identified nucleotide sequence reagents have been identified across several miRNA knowledgebases (56), indicating that unreliable gene research publications are already affecting biocuration and text mining. Where problematic papers contribute substantially to the literature on individual genes, these genes could be wrongly prioritised by translational studies (27), leading to further research waste. For example, problematic preclinical gene research could lead to the incorrect selection of candidate disease biomarkers that may be more likely to fail to progress to clinical application (27).

## IMPROVED AWARENESS AND DETECTION OF PROBLEMATIC GENE RESEARCH

While publishers and individual journals are becoming increasingly aware of paper mills (8,10,32,36,62–73), this awareness is unlikely to extend to researchers, who generally expect low rates of outright research fraud (7,8). Reasonable expectations that most published research derives from genuine efforts, combined with limited and fragmented discussions of paper mills, are likely to result in most researchers who carry out, review, and/or translate preclinical human gene research being unaware that manuscripts and publications could derive from paper mills.

An important and urgent first step is to raise the awareness of problematic gene research papers and paper mills within the gene research community. Institutionally-supported education that provides information about features of problematic research and paper mills (Table 2) will allow trainees and researchers to more critically assess the literature that they use to inform their projects and experiments (8,10,27,32). An awareness of problematic research will encourage researchers to take simple steps such as assessing research justifications for plausibility and checking manuscripts and publications for other features associated with paper mills and/or poor-quality research (Table 2).

In the experimental sciences, trainee education sessions should include open discussions about failed replication experiments, to provide specific reassurance that failure to reproduce published results does not necessarily reflect individual research skill or aptitude (80). The knowledge that published gene research might be unreliable should also inform the design of replication experiments, such that these require the routine checking of verifiable reagent identities before experiments commence, pre-defined replication stop-points and/or steps to move failed replication attempts away from trainees towards senior laboratory staff.

Just as academic institutions should provide education about paper mills, all publishers should ensure that editors, peer reviewers and journal staff are provided with regularly-updated information about the features of manuscripts from paper mills. As many features of problematic gene research benefit from expert knowledge for their detection (Table 2), journals and publishers should specifically prompt editors and peer reviewers to consider whether manuscripts have features that could reflect paper mill involvement. One repeatedly noted feature of suspected paper mill manuscripts and articles is the absence of mechanistic hypotheses to link gene(s), systems, and experimental approaches through novelty-based research justifications (10,65,71). Laboratory scientists and students should easily recognize the implausibility of conducting extensive suites of experiments (Figure 2) simply because genes have never been examined in particular biological or clinical contexts. Given the well-recognized challenges of generating reviewer-requested data (81), editors and peer reviewers should also ensure that revisions to gene research manuscripts do not require gratuitous data that could be more easily generated by paper mills than through genuine research.

## DETERRING FUTURE SUBMISSIONS FROM PAPER MILLS

In addition to detecting manuscripts from paper mills, some journals have described methods to deter future paper mill submissions. These approaches have mostly focused upon incremental changes to manuscript standards (36,64,70,72,82). However, approaches such as specifying the numbers of gene knockdown reagents to be used in experiments (64), requiring authors to employ ORCID identifiers (72,82) or include declarations that ‘no paper mill was used’ (36,82) have either not proved effective in other settings (83), or can be very easily addressed or gamed by paper mills (32,84). Rather than deterring submissions, incremental changes to manuscript standards could enable the production of more plausible manuscripts that could be accepted by more journals. There is some evidence that paper mills can switch publication topics and templates in response to detection (85,86), and new developments in artificial intelligence (73,87,88) can allow the invention of unique images that may not be flagged as problematic.

As paper mills could easily scale the production of superficial, novelty-driven gene research manuscripts with claimed relevance to disease (Figure 4), journal requirements that insist on focussed research justifications and technical approaches could provide some level of deterrence (10,71,89–92), at least in the short-term. In-depth mechanistic analyses may be less capable of generating broad claims of clinical relevance and may therefore be less valued by some paper mill clients, and less plausibly conducted in particular settings. As paper mills have likely generated a broad base of literature upon which research justifications could be based (32,56), such as the analysis of particular genes in a new cancer type, journals should also require gene research topics to be supported by multiple triangulating sources of evidence (93). These sources must not represent multiple poorly-justified studies that could themselves originate from paper mills (56) (Table 2).

Given the capacity of paper mill submissions to evolve, deterring paper mill submissions through specific manuscript requirements is likely to be ineffective in the long term (60). The research and publishing communities should also consider approaches that target the rate-limiting steps for fraudulent versus genuine research (Figure 2). In contrast to genuine gene research that can require years to complete, the current rate-limiting step for paper mills is likely to represent the peer review process (Figure 2). The value of accelerating this rate-limiting step has undoubtedly been recognized by paper mills, through strategies such as editorial and peer review manipulation, and submissions of the same or very similar manuscripts to multiple journals (8,10,32,33,35,63,73).

Fabricated gene research manuscripts could be deterred by introducing new rate-limiting steps that specifically target paper mills. One method to selectively delay manuscript submissions by paper mills could be the registration of human gene research prior to submission (Table 3). Gene research registration could differ from other forms of study registration (95,96), by occurring at any research stage, not involving peer review, but simply conferring the future capacity to submit a specified manuscript as defined by its title, abstract and authors. The key feature of gene research registration would be to require minimum time periods between registration and manuscript submission, which would be chosen to align with and support the requirements of genuine experimental research (Figure 2). The requirement to specify both study topic and authors in advance of manuscript submission would be highly unfavorable to paper mills, where manuscript construction and author identification are likely to be separate activities (8,9,32,33).

**Table 3.** Approaches to deter human gene research manuscripts and/or publications from paper mills

Proposed scheme (references)	Description	Intended effects on paper mills	Intended effects on genuine research	Possible challenges and/or unintended consequences
Study registration (94,95)	• Adaptation of study registration for gene research • Journals could recommend that gene research studies be registered prior to manuscript submission	• Delayed submissions from paper mills, by increasing time from manuscript production to submission • Reduced targeting of gene research by paper mills	• Reduced time advantage of fraudulent gene research compared with genuine research	• Registration requirements would need to reflect time-scales of genuine gene research • Registration would need to be co-designed with gene researchers to ensure minimal impact on genuine research • Registration system would require funding and support
Preprints (10,73)	• Require manuscripts to be posted to preprint server at time of submission • Preprints to be clearly identified with journal and date of submission	• Reduced duplicate manuscript submissions from paper mills	• Reduced waste of editorial and peer reviewer time	• Paper mill submissions could shift to journals that do not require preprints • Cross-publisher support would be required to be effective
Editorial notes and/or expressions of concern (10)	• Papers with verifiable errors (wrongly identified nucleotide sequences, cell lines) to be rapidly and transparently flagged by indexed error notifications	• Quicker flagging of problematic papers • Improved capacity to correct or remove publications with verifiable errors	• Increased awareness of publications with errors • Reduced attention to and citations of problematic research	• Restricted to papers with objective, verifiable errors • Could drive evolution of paper mill manuscripts towards: • manuscripts with fewer/ no errors • topics that lack verifiable reagents

At the time of manuscript submission, paper mills can increase manuscript acceptance rates by simply submitting the same manuscripts to multiple journals, which is highly wasteful of editorial and peer review resources (10,32,36,72). As a result, through the STM collaboration, publishers are now sharing information to reduce duplicate submissions of identical or very similar manuscripts (32). We and others have also recommended that journals mandate preprint posting of gene research and other manuscripts (10,72,73) (Table 3). Requirements to preprint manuscripts where both the submission date and journal are clearly displayed (Table 3) could reduce the burden of duplicate submissions on journals and peer reviewers (10,72), and the numbers of paper mill manuscripts that are accepted for publication.

Paper mills will also be selectively disadvantaged by geometrically increasing the rate and scale of post-publication corrections (10) (Table 3). Indeed, the recent finding that most retractions occur when research attention has been exhausted highlights the need for more rapid responses to post-publication concerns (96). Due to the time required to achieve published responses to error notifications (39), it has been proposed that papers with verifiable errors should be flagged within the literature when journal investigations commence, as opposed to when investigations conclude (10,53) (Table 3). The identification of verifiable errors such as wrongly identified nucleotide sequences (38,39,54,56) or misidentified or contaminated cell line models (97) could immediately precipitate the publication of an indexed editorial note (10) or expression of concern. The knowledge that journals will immediately flag verifiable published errors could also encourage more researchers to report verifiable published errors.

## THE CASE FOR FUTURE RESEARCH

To date, manuscripts or articles that may have been produced with assistance from paper mills has been recognised by researchers analysing unexpected literature trends (38,85,86) and/or verifiable errors (38,39,56) and by journal editors and experts describing unusual manuscript or publication features (10,36,37,62–73,82). Others have undertaken the challenging task of interviewing authors who may have engaged with paper mills (35) or obtaining information directly from suspected paper mills (9,42). While recognising the difficulties in studying covert activities, the research conducted has been retrospective, and some evidence is now dated.

The limited research conducted to date highlights the need to fill many outstanding knowledge gaps about paper mill products and services (11). Research is urgently needed to produce a more comprehensive description of the features of paper mill-supported articles, how these features may be changing over time, and of the extent to which paper mills have contributed to the gene research and other biomedical literature. Similarly, research is urgently required to inform open questions such as when paper mills began to target human genes as research topics, whether genes in model organisms have also been targeted, and how features of problematic gene research papers are changing over time. Analyses of database indexing and citations of problematic gene research papers are also required to demonstrate how these publications may impact future research (37,56).

Other forms of research can render genes more resistant to systematic fraud (54). Funding agencies could support the investigation of more individual genes and proteins (98–100). A larger community of researchers that focuses on less well-characterized genes could yield valuable new biology insights, while also creating safeguards such as expert peer review communities, as well as data for information triangulation and fact-checking (27,54). Funding bodies can also support community initiatives to improve the quality of gene research, such as data integration to permit efficient leveraging of large-scale datasets (100), to retrospectively determine whether claims from gene-centric publications generalize to other experimental studies, and/or are supported by high-throughput approaches. Gene information portals and knowledgebases need to be adequately resourced, both to promote researcher awareness and use (100), and to ensure timely updates in response to post-publication corrections and retractions.

## SUMMARY AND CONCLUSIONS

Publish or perish research cultures (6,34,43,101) combined with an increasingly commercially-focused publishing environment (49,102) are leading to the dislocation between the scientific and career value of research publications (103), where human genes may provide unparalleled opportunities for systematic research fraud (Figure 4). The availability of ∼40 000 human genes that can be plausibly studied singly and in combination using widely employed, accessible experimental techniques across different biological and disease contexts could allow scaled manuscript production to match the requirements of large author populations who are experiencing unreasonable pressures to publish (Figure 4) (27).

Through their capacity to be produced at scale, fraudulent gene research manuscripts and publications can waste publisher, journal and research resources, damage biomedical research careers at all stages, and devalue the contributions of human subjects and animal models to preclinical gene research. Widespread fraudulent gene research will encourage the financial support of unproductive research directions, slow research translation through opportunity costs, and reduce confidence in research and the scientific method (10,33,56). Without effective interventions, paper mill contributions will continue to grow, generating more papers across many research fields and journals, and reaching more researchers through an expanding body of literature citations. Any failure to act against paper mills will eventually result in the loss of trust in large swathes of the human gene research literature and could result in researchers individually or collectively abandoning important research fields.

Academic and public institutions have major roles to play in dismantling the range of perverse incentives that are likely to drive clients towards paper mills, including publication quotas, career rewards for publication numbers, and cash publication bonuses (32,48,82,101). Similarly, publishers and journals have responsibilities to not only detect problematic manuscripts (32), but to also urgently increase the removal of incorrect information from the published record that is being used to inform research (39). In the meantime, education and training opportunities for trainees and researchers, including those who serve as journal editors and peer reviewers, are needed to raise much-needed awareness of problematic gene research publications. A broader awareness of problematic manuscript and publication features (Table 2) (8,32,104) can help gene researchers, editors and peer reviewers to prioritise more reliable information sources that are supported by plausible research justifications and rigorous and transparently described experimental approaches. By approaching the gene research literature with a more critical mindset, researchers can avoid the costly, time-wasting and misleading traps of targeted gene research produced without experiments.

## DATA AVAILABILITY

No new data were generated or analysed in support of this research.
